# Editorial: Moonshot goals of soft robotics through embodied intelligence

**DOI:** 10.3389/frobt.2026.1929066

**Published:** 2026-08-19

**Authors:** Nana Obayashi

**Affiliations:** Prema Lab, Center for Robotics and Embodied Intelligence, New York University, Brooklyn, NY, United States

**Keywords:** bio-inspired design, embodied intelligence, morphological computation, reproducibility, soft robotics

Soft robotics has redefined traditional robotics. By introducing fully compliant, bio-inspired designs capable of thriving in diverse environments, the field has demonstrated that bodies which bend, deform, and adapt passively can achieve tasks well beyond the reach of rigid systems. Since its rise in the 2010s, soft robotics has quickly progressed, and yet research in recent years has shown mainly incremental advances, with open questions to fundamental challenges remaining. To avoid stagnation and continue positive momentum, the field must define a bold vision for the future.

This Research Topic takes up that challenge. Focusing on embodied intelligence—the dynamic interplay between body, brain, and environment—we sought to unite researchers from within and beyond the field to establish both short-term (5-year) and long-term (10- to 20-year) moonshot goals. The aim was to create an “Apollo moment” for the soft robotics community: a shared, ambitious, and inclusive vision that propels the field into the next technological wave. Our goals were twofold. First, to define a path for sustainable growth, ensuring soft robotics becomes a lasting and impactful research area capable of addressing major global challenges beyond the scope of traditional robotics. Second, to promote the democratization of the field, thereby fostering interdisciplinary collaboration, expanding access, and reshaping the structures that determine who participates and how.

The four contributions collected here each engage with these goals from a different angle.

Howard’s perspective on reproducibility addresses a challenge the field has largely avoided naming directly: that soft robotics, precisely because it is experimental and material-dependent, is exceptionally difficult to replicate. Reproducibility is the mechanism by which results accumulate into knowledge, and a prerequisite for sustainable growth. Howard’s three moonshot goals in this space, covering standardized data sharing, cross-disciplinary testing protocols, and quantitative comparison of embodied intelligence, define a concrete path toward a field that can build on itself rather than repeatedly starting from scratch.

Chan and Scharff’s mini-review on passive adaptive grippers grounds the abstractions of embodied intelligence in a well-defined design problem. Grippers that leverage interaction between the gripper and the environment rather than active control to achieve prehension, retention, and release are among the clearest demonstrations of what bio-inspired, fully compliant design can offer. Their classification framework and identification of remaining trade-offs, particularly the tension between controllability and host flexibility, map the open engineering questions that the next wave of soft robotics research will need to address.

The contribution from Vihmar et al. introduces a robot capable of spinning fibrous structures *in situ* by exploiting environmental dynamics directly. The work is a concrete implementation of embodied intelligence principles that the field has often invoked but rarely demonstrated at this level: a robot that extends, repairs, and reconfigures its own physical architecture in response to changing conditions, incorporating environmental information through interaction rather than sensing it from a distance. This spiderweb-inspired approach points toward robots that are not just adaptable, but generative which is capable of expanding their capabilities beyond their initial physical architecture.

Finally, Wakatsuki and Yamada examine whether human motor control shows behavioral signatures consistent with morphological computation, the idea that body mechanics themselves contribute to computation under uncertainty. This is a proof-of-concept study, and the authors are appropriately careful about what can be claimed from a single-subject design. But its inclusion reflects a core commitment of this Research Topic: that the bold vision for soft robotics must remain in active dialogue with biology and neuroscience, and that the diverse voices needed to define an Apollo moment for the field extend well beyond robotics as traditionally conceived.

These contributions demonstrate that we will require the kind of interdisciplinary collaboration and inclusive, expansive thinking this Research Topic was designed to encourage.

